# Sensing and Connection Systems for Assisted and Autonomous Driving and Unmanned Vehicles

**DOI:** 10.3390/s18071999

**Published:** 2018-06-22

**Authors:** Sergio Saponara

**Affiliations:** Dipartimento di Ingegneria della Informazione, Università di Pisa, via G. Caruso 16, 56122 Pisa, Italy; sergio.saponara@unipi.it

**Keywords:** Unmanned Aerial/Underwater/Ground Vehicles (UAV/UU/UGV), assisted and autonomous driving, sensors, cooperative mobility, path planning, driver and passenger health status monitoring, vehicular networks, cybersecurity, low-latency, embedded systems

## Abstract

The special issue, “Sensors, Wireless Connectivity and Systems for Autonomous Vehicles and Smart Mobility” on MDPI Sensors presents 12 accepted papers, with authors from North America, Asia, Europe and Australia, related to the emerging trends in sensing and navigation systems (i.e., sensors plus related signal processing and understanding techniques in multi-agent and cooperating scenarios) for autonomous vehicles, including also unmanned aerial and underwater ones.

## 1. Introduction

The proposed special issue has invited submissions related to sensing and connectivity systems for assisted and autonomous driving and for unmanned aerial, underwater and ground vehicles (namely UAV, UUV and UGV) [1,2,3,4,5,6,7,8,9,10,11,12]. Recent studies on the future of the automotive and robotic industry predict that the borders between the ICT (Information and Communication Technology) industry and the consumer industry will blur, and that vehicles will become consumer-centric [13]. The huge market of 90-million vehicles sold worldwide per year is suited for integrated electronics and MEMS/MOEMS (micro electro/opto-electro mechanical systems) technologies and embedded control and signal processing tasks. Indeed, around 80% of all innovations in new vehicles are driven by electronics and sensor technology to implement green and safe vehicles for sustainable and smart mobility. The value in vehicles is shifting from chassis and powertrain to electronics and sensors, which will cover one-third of a car’s cost in 2025. Lots of sensing and communication components, including wireless devices, can be used on-board vehicles. However, vehicular electronics require high performance components, must be able to operate in harsh environments, and must ensure fault robustness and functional safety. Most devices have originally been developed for consumer applications, and their use for autonomous driving is not straightforward. 

The particular topics of interest, covered by the 12 papers published in this special issue, are:

- a cooperative search and coverage algorithm in a scenario with several unknown targets, by a team of unmanned vehicles;
- a cooperative navigation algorithm for multiple unmanned vehicles considering communication delay, efficient formation control method and initialization errors;
- trajectory control for teleoperated robots and vehicles;
- vehicular networks for safe and secure smart mobility;
- biometric sensors to measure driver and passenger health and fatigue and attention status for safety and security;
- sensing systems for the precise localization of vehicles.

All of the published works present experimental results to validate the proposed techniques.

## 2. Review of the Contributions in the Special Issue

The issue of cooperative algorithms for unmanned aerial, underwater or ground vehicles (UAV/UUV/UGV) is addressed in half of the works that are published in the special issues [1,2,3,4,5,6], with the first four from Universities in China and the last two from South Korea and Spain. 

More in detail, Ref. [1] presents a cooperative search and coverage algorithm for a given bounded rectangle region, which contains several unknown stationary targets, by a team of UAVs with non-ideal sensors and limited communication ranges. The goal of the proposed cooperative search and coverage algorithm is minimizing the search time, while gathering more information about the environment and finding more targets. Firstly, as the representation of the environment, the cognitive maps that included the target probability map (TPM), the uncertain map (UM) and the digital pheromone map (DPM) are constituted. Secondly, a controllable revisit mechanism based on the DPM is designed. This mechanism can concentrate the UAVs to revisit sub-areas that have a large target probability or high uncertainty. Thirdly, a path planning algorithm for the multi-UAV’s cooperative search and coverage is designed. In the path planning algorithm, the movement of the UAVs is restricted by the potential fields to meet the requirements of avoiding collision and maintaining connectivity constraints. Moreover, using the minimum spanning tree (MST) topology optimization strategy, a tradeoff between the search coverage enhancement and the connectivity maintenance is obtained. The feasibility of the proposed algorithm is demonstrated by comparison simulations by way of analyzing the effects of the controllable revisit mechanism and the connectivity maintenance scheme. The Monte Carlo method is employed to validate the influence of the number of UAVs, the sensing radius, the detection and false alarm probabilities and the communication range on the proposed algorithm. 

Technologies for UUV are addressed in Refs. [2,3,4], which are emerging as mobile sensor networks for the investigation and exploration of the underwater environment. Particularly, Ref. [2] proposes a polar cooperative navigation algorithm for multi-UUVs considering communication delays to solve the navigation accuracy problems of multi-Unmanned Underwater Vehicles (multi-UUVs) in the polar region. For UUVs to complete missions, precise navigation is necessary. It is difficult for UUVs to establish true headings because of the rapid convergence of Earth meridians and the severe polar environment. Based on the polar grid navigation algorithm, UUV navigation in the polar region can be accomplished with the Strapdown Inertial Navigation System (SINS) in the grid frame. To save costs, a leader-follower type of system is introduced in this paper. The leader UUV helps the follower UUVs to achieve high navigation accuracy. Follower UUVs correct their own states based on the information sent by the leader UUV and the relative position measured by ultra-short baseline acoustic positioning. The underwater acoustic communication delay is quantized by the model. In Ref. [2], considering the underwater acoustic communication delay, the conventional adaptive Kalman filter (AKF) is modified to adapt to polar cooperative navigation. The results demonstrate that the polar cooperative navigation algorithm for multi-UUVs that considers communication delays can effectively navigate the sailing of multi-UUVs in the polar region. Instead, in Ref. [3] the target is to develop a practical and efficient formation control method to improve the work efficiency of multi-UUV sensor networks. Distributed leader-follower formation controllers are designed based on a state feedback and consensus algorithm. Considering that each vehicle is subject to model uncertainties and current disturbances, a second-order integral UUV model with a nonlinear function is established using the state feedback linearized method under current disturbances. For unstable communication among the UUVs, communication failure and acoustic link noise interference are considered. Two-layer random switching communication topologies are proposed to solve the problem of communication failure. For acoustic link noise interference, the accurate representation of valid communication information and noise stripping when designing controllers is necessary. Effective communication topology weights are designed to represent the validity of the communication information interfered by noise. Utilizing state feedback and noise stripping, sufficient conditions for design formation controllers are proposed to ensure that UUV formation achieves consensus under model uncertainties, current disturbances and unstable communication. The stability of the formation controllers is proven by the Lyapunov-Razumikhin theorem, and the validity is verified by simulation results.

Initial alignment is crucial in UUV because the initial alignment results will be used as the initial SINS value, which might affect the subsequent SINS results. Due to the rapid convergence of Earth meridians, there is a calculation overflow in conventional initial alignment algorithms, making conventional initial algorithms invalid for polar UUV navigation. To overcome these problems, a polar initial alignment algorithm for UUV is proposed in Ref. [4], which consists of coarse and fine alignment algorithms. Based on the principle of the conical slow drift of gravity, the coarse alignment algorithm is derived under the grid frame. By choosing the velocity and attitude as the measurement, the fine alignment with the KF is derived under the grid frame. Simulation and experiment are realized among polar, conventional and transversal initial alignment algorithms for polar UUV navigation. The results demonstrate that the proposed polar initial alignment algorithm can complete the initial alignment of UUV in the polar region rapidly and accurately.

Ref. [5] presents a distributed coordination methodology for multi-robot systems, based on nearest-neighbor interactions. Among many interesting tasks that may be performed using swarm robots, Ref. [5] proposes a biologically-inspired control law for a shepherding task, whereby a group of external agents drives another group of agents to a desired location. First, sheep-like robots that act like a flock are generated. The assumption is that each agent is capable of measuring the relative location and velocity to each of its neighbors within a limited sensing area. Then, a control strategy is designed for shepherd-like robots that have information regarding where to go and a steering ability to control the flock, according to the robots’ position relative to the flock. Several independent behavior rules are defined. Each agent calculates to what extent it will move by summarizing each rule. The flocking sheep agents detect the steering agents and try to avoid them, and this tendency leads to the movement of the flock. Each steering agent only needs to focus on guiding the nearest flocking agent to the desired location. Without centralized coordination, multiple steering agents produce an arc formation to control the flock effectively. In addition, a new rule for collecting behavior is proposed, whereby a scattered flock or multiple flocks are consolidated. From simulation results with multiple robots, Ref. [5] shows that each robot performs actions for the shepherding behavior, and only a few steering agents are needed to control the whole flock. The results are displayed in maps that trace the paths of the flock and steering robots. Performance is evaluated via time cost and path accuracy to demonstrate the effectiveness of this approach.

Ref. [6] describes the theoretical and practical foundations for the remote control of a mobile robot for nonlinear trajectory tracking using an external localization sensor. It constitutes a classical networked control system, whereby event-based techniques for both control and state estimation contribute to the efficient use of communications and reduce sensor activity. Measurement requests are dictated by an event-based state estimator by setting an upper bound to the estimation error covariance matrix. The rest of the time, state prediction is carried out with the unscented transformation. This prediction method makes it possible to select the appropriate instants at which to perform actuations on the robot so that guidance performance does not degrade below a certain threshold. Ultimately, we obtained a combined event-based control and estimation solution that drastically reduces communication accesses. The magnitude of this reduction is set according to the tracking error margin of a P3-DX robot following a nonlinear trajectory that is remotely controlled with a mini PC, and whose pose is detected by a camera sensor.

One of the main issues in smart vehicles is monitoring the health and attention status of the drivers and the passengers for both comfort and safety issues. To this aim, Ref. [7] from Virginia Tech, USA, demonstrates the feasibility of using a seat sensor designed for occupant classification from a production passenger vehicle to measure an occupant’s respiration rate (RR) and heart rate (HR). Relaying occupant vital signs after a crash could improve emergency response by adding a direct measure of the occupant state to an Advanced Automatic Collision Notification (AACN) system. Data were collected using a Ford Mustang passenger seat and seat sensor. 

Vehicular ad hoc networks (VANET) for safe and secure smart vehicles under severe latency constraints are addressed in Ref. [8], from a joint research group from Australia and Thailand. In high-density road networks, with each vehicle broadcasting multiple messages per second, the arrival rate of safety messages can exceed the rate at which digital signatures can be verified. Unfortunately, not all messages can be verified. Hence, to ensure that each vehicle receives the appropriate awareness of neighboring vehicles, algorithms for selecting which messages to verify are required. To this aim, Ref. [8] presents a novel scheme to select important safety messages for verification in VANETs, by using the location and the direction of the sender, as well as the proximity and relative-time between vehicles to reduce the number of irrelevant messages that are verified (i.e., messages from vehicles that are unlikely to cause an accident). Compared with other existing schemes, the analysis results show that the proposed scheme can verify messages from nearby vehicles with lower inter-message delay and reduced packet loss, and thus it provides a high level of awareness of the nearby vehicles.

Ref. [9], from Universidad Carlos III in Madrid, Spain, presents an architecture to carry out the estimation and control of vehicle dynamics, which integrates low-cost sensors and embedded hardware. A comparison in terms of accuracy, acquisition time and reliability vs. commercial products, VBOX device from Racelogic, has been made from tests carried out in a real vehicle. 

Ref. [10] from France addresses the problem of visual-based localization in seasonal changing situations, which is one of the most challenging topics in computer vision and the intelligent vehicle community. The difficulty of this task is related to the strong appearance changes that occur in scenes due to weather or season changes. In Ref. [10], a place recognition based visual localization method is proposed, which realizes the localization by identifying previously visited places using the sequence matching method. It operates by matching query image sequences to an image database that has been acquired previously (video acquired during the traveling period). In this method, in order to improve matching accuracy, multi-feature is constructed by combining a global GIST descriptor and local binary feature CSLBP (Center-symmetric local binary patterns) to represent image sequence. Then, similarity measurement according to Chi-square distance is used for effective sequence matching. For experimental evaluation, the relationship between the image sequence length and the sequence’s matching performance is studied. To show its effectiveness, the proposed method is tested and evaluated in four season’s outdoor environments. The results have shown improved precision–recall performance against the state-of-the-art SeqSLAM algorithm.

Ref. [11], still from a Chinese University, presents a four-layer Connected and Autonomous Vehicles (CAVs) kinematic simulation framework, which is composed with a road network layer, a vehicle operating layer, an uncertainties modelling layer and a demonstrating layer. The properties of the intersections are defined to describe the road network. A target position-based vehicle position updating method is designed to simulate vehicle behaviors, such as lane changing and turning. Vehicle kinematic models are implemented to maintain the status of the vehicles when they are moving towards the target position. Priorities for individual vehicle control are authorized for different layers. The operation mechanisms of the CAV’s uncertainties, which are defined as position error and communication delay in this paper, are implemented in the simulation to enhance the reality of the simulation. A simulation platform is developed based on the proposed methodology. A comparison of the simulated and theoretical vehicle delay has been analyzed to prove the validity and the creditability of the platform. The scenario of rear-end collision avoidance is conducted to verify the uncertainties operating mechanisms, and a slot-based intersections control strategy is realized and verified in the simulation platform to show the supports of the platform to CAVs kinematic simulation and verification.

Ref. [12], from a collaboration between a Germany and Chinese University and a UK company, addresses the problem of precise localization, which is a key requirement for the success of assisted and autonomous vehicles. The diminishing cost of hardware has resulted in a proliferation of the number of sensors in the environment. Cooperative localization (CL) presents itself as a feasible and effective solution for localizing the ego-vehicle and its neighboring vehicles. However, one of the major challenges to fully realize the effective use of infrastructure sensors for jointly estimating the state of a vehicle in cooperative vehicle-infrastructure localization is an effective data association. Ref. [12] proposes a method which implements symmetric measurement equations within factor graphs in order to overcome the data association challenge with a reduced bandwidth overhead. The simulated results demonstrate the benefits of the proposed approach in comparison with a previously proposed approach of topology factors. 

## 3. Conclusions

The proposed special issue, “Sensors, Wireless Connectivity and Systems for Autonomous Vehicles and Smart Mobility” on MDPI Sensors has presented 12 works, from authors operating in US, China, South Korea, Spain, UK, Germany and Australia. The presented works are related to the emerging trends in sensing systems (i.e., sensors plus related signal processing and understanding techniques in multi-agent and cooperating scenarios) for autonomous vehicles, including also unmanned aerial and underwater ones. All the published works present experimental results to validate the proposed techniques.
